# Tracking the Genetic Susceptibility Background of B-Cell Non-Hodgkin’s Lymphomas from Genome-Wide Association Studies

**DOI:** 10.3390/ijms22010122

**Published:** 2020-12-24

**Authors:** Isaias Hernández-Verdin, Karim Labreche, Marion Benazra, Karima Mokhtari, Khê Hoang-Xuan, Agusti Alentorn

**Affiliations:** 1 Faculté de Médecine, Sorbonne Université, 75013 Paris, France; isaias.hernandez@icm-institute.org (I.H.-V.); karim.labreche@icm-institute.org (K.L.); marion.benazra@icm-institute.org (M.B.); khe.hoang-xuan@aphp.fr (K.H.-X.); 2 Brain and Spine Institute (ICM), 75013 Paris, France; 3 National Institute of Health and Medical Research (Inserm) U 1127, 75013 Paris, France; 4 National Center for Scientific Research, Joint Research Unit 7225, 75013 Paris, France; 5 Brain and Spine Institute (ICM), iGenSeq Platform, 75013 Paris, France; 6 Raymond Escourolle Department of Neuropathology, Public Assistance–Hospitals of Paris, Hospital Group of Pitié-Salpêtrière, 75013 Paris, France; karima.mokhtari@aphp.fr; 7 Assistance Publique Hôpitaux de Paris (APHP), Department of Neurology-2, Groupe Hospitalier Pitié Salpêtrière, 75013 Paris, France; 8 Réseau Expert National LOC (Lymphomes Oculo-Cérébraux), Groupe Hospitalier Pitié Salpêtrière, 75013 Paris, France

**Keywords:** B-cell non-Hodgkin’s lymphoma, GWAS, cancer risk, HLA

## Abstract

B-cell non-Hodgkin’s lymphoma (NHL) risk associations had been mainly attributed to family history of the disease, inflammation, and immune components including human leukocyte antigen (HLA) genetic variations. Nevertheless, a broad range of genome-wide association studies (GWAS) have shed light into the identification of several genetic variants presumptively associated with B-cell NHL etiologies, survival or shared genetic risk with other diseases. The present review aims to overview HLA structure and diversity and summarize the evidence of genetic variations, by GWAS, on five NHL subtypes (diffuse large B-cell lymphoma DLBCL, follicular lymphoma FL, chronic lymphocytic leukemia CLL, marginal zone lymphoma MZL, and primary central nervous system lymphoma PCNSL). Evidence indicates that the HLA zygosity status in B-cell NHL might promote immune escape and that genome-wide significance variants can give biological insight but also potential therapeutic markers such as WEE1 in DLBCL. However, additional studies are needed, especially for non-DLBCL, to replicate the associations found to date.

## 1. Introduction

Malignant lymphomas are among the most common head and neck neoplasm from lymphoreticular system origin and can be defined as Hodgkin’s or non-Hodgkin’s lymphoma (NHL), in which approximately 25% arises from extra-nodal locations like Waldeyer’s ring, oral cavity, salivary glands, thyroid, larynx, nasal cavity, paranasal sinuses, skin, brain, eyes, leptomeninges, or spinal cord [1,2]. According to the 2016 World Health Organization classification there are around 60 distinct subtypes of NHL with diffuse large B-cell lymphoma (DLBCL, about 30%), follicular lymphoma (FL, about 20%) and chronic lymphocytic leukemia/small lymphocytic leukemia (CLL/SLL) among the most common [3,4,5]. Along with the wide location distribution of the lymphomas, this group of diseases has varying etiologies and prognosis, for example the five-year survival is 85% for CLL, 80% for FL, 76.5% for marginal zone lymphoma (MZL), but <50% for more aggressive lymphomas such as DLBCL or even 30% for primary central nervous system lymphoma (PCNSL) [6,7,8]. Furthermore, there have been vast population studies to associate the presence of different etiologies, such as smoking, height, weight, autoimmune conditions, alcohol consumption, viral infections, and genetics, with the risk of developing any subtype of NHL [3,9,10]. Nevertheless, despite these efforts, there are only few established risk factors including autoimmune conditions (e.g., Sjögren disease, rheumatoid arthritis, systemic lupus erythematosus, and multiple sclerosis), immunodeficiency syndromes, organ transplants, breast implants and specific infections (e.g., *Helicobacter pylori* for mucosa-associated lymphoid tissue lymphoma of the stomach, immunodeficiency virus, and mononucleosis) [11,12,13].

More recently, sequencing technologies like next generation sequencing (NGS) and genome-wide association studies (GWAS), have broaden the possible candidates by using thousands of genetic variants for multiple genetic risk factors identification [14]. GWAS combining the population structure (Q) jointly with the genetic marker based kinship matrix (K) mixed linear model, also called linear mixed model, where the test statistic for significance is drawn from the central Chi-square distribution by comparing the allele frequencies of the cases to the controls. A variant is said to be significant at genome-wide level if the *p* value is ≤5 × 10^−8^, which was set by taking a 0.05 significance level and roughly dividing by the total number of independent blocks of linked genes in Europeans (thought to be 1,000,000) [7,15]. Regarding GWAS within the B-cell NHL context, most studies have focused on genetic variants at chromosome 6p21, specifically human leukocyte antigen (HLA) variants, since that region is critical for innate and adaptive immune responses, but there have been also efforts to find associations with variants outside this chromosome and other etiologies [7,10,11,16,17,18,19].

In this review, we give an overview of HLA structure and diversity, and then we summarize the most recent GWAS presented in five B-cell NHL subtypes: DLBCL, FL, CLL, MZL, and PCNSL in the light of loci (HLA and others) diversity and zygosity specific associations in addition to further clinical evaluations when suitable. Furthermore, we present the genetic overlap between B-cell NHL subtypes and autoimmune diseases, height, lipid traits, or other lymphomas.

## 2. HLA Overview

The association found between Hodgkin’s lymphoma (HL) and HLA-B gene variation allowed to the later discovery that the major histocompatibility complex (MHC) is the genomic region with the highest number of associated human diseases [20,21]. The MHC, a hyper gene-dense region located at chromosome 6p21.3, encodes a set of 21 protein-coding loci that gives rise to three different types of HLA molecules. Firstly, class I, encoded by the highly polymorphic *HLA-A*, *HLA-B,* and *HLA-C* genes (“classical”); but also *HLA-E*, *HLA-F,* and *HLA-G* genes (“nonclassical”) both of which are composed of a single α chain non-covalently bound to a small β2-microglobulin polypeptide encoded by another chromosome (15q21). Secondly, class II, encoded by the *HLA-DPA1*, *HLA-DQA1*, *HLA-DQA2*, *HLA-DRA*, *HLA-DPB1*, *HLA-DQB1*, *HLA-DQB2*, *HLA-DRB1*, *HLA-DRB2*, *HLA-DRB3*, *HLA-DRB4,* and *HLA-DRB5* genes which are composed of α-β heterodimer. Thirdly, class III, encoded by 61 genes (ex. *MIC, SKI2W*) involved in inflammation, leukocyte maturation and complement cascade [7,22,23,24]. These HLA genes comprise approximately four million base pair region, giving rise to more than 15,000 different classical HLA class I and II alleles which can, theoretically, serve for presenting over 10^12^ different peptides if the antigen-presenting cell (APC) is heterozygous at each of the six classical class I or II HLA loci [25,26].

**Figure 1:**
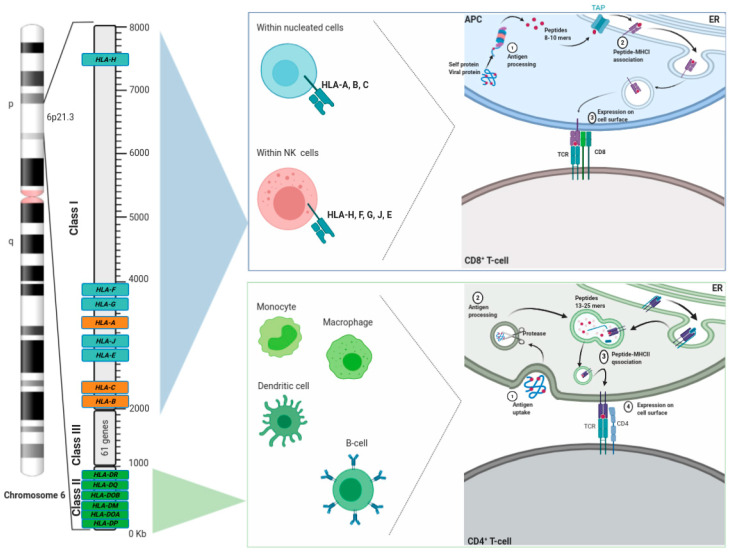
Schematic map of the human leukocyte antigen (HLA) genomic region showing the distribution of HLA genes along with the summarized mechanism of antigen presentation.

Nucleated cells express HLA class I molecules where small peptides (8–10 amino acids) are bound to the α1-α2 domains at the HLA peptide-binding site for later recognition by αβ T-cell receptors (TCRs) on CD8+ T cells. On the other hand, monocytes/macrophages, dendritic cells and B cells express HLA class II receptors which can present larger peptides (13–25 amino acids) to TCRs on CD4+ T cells, hence inducing the orchestrated immune response against the pathogen due to cytokines releasing either by helping B cells to secrete high affinity antibodies or by inducing macrophage activation [7,22,23]. Furthermore, HLA class I molecules can bind to natural killer (NK) cells through immunoglobulin-like receptors and C-type lectin-like CD94/NKG2 receptors (Figure 1) [27].

Along with the extreme gene density and polymorphism at the MHC locus, linkage disequilibrium, low-throughput methodologies and samples sizes made HLA-disease associations complicated. However, with the advent of both high-throughput whole-genome-based methodologies (example GWAS) and the evolution of big data analysis, researchers can measure the contribution of a single genetic variation across the genome on a disease risk by leveraging linkage disequilibrium [15].

## 3. GWAS in B-Cell NHL

### 3.1. DLBCL

Representing around 30% of NHL and affecting preferentially older adults, diffuse Large B-cell lymphoma is the most common type of NHL. During the last two decades, treatment with immunochemotherapy consisting of cyclophosphamide, doxorubicin, vincristine, and prednisolone combined with the anti-CD20 monoclonal antibody rituximab (R-CHOP) has become the gold standard. This regimen results in a cure rate of 60% and a five-year survival rate of 60% for germinal center B-cell (GCB) subtype or 35% for activated B-cell (ABC) subtype; however, clinical course is heterogeneous, even after elucidation of cell of origin (ABC or GCB), thus requiring new biomarkers for elucidating patient outcome and for adapting treatment strategies [28,29]. Different risks factors have been identified. A stepwise logistic regression meta-analysis of 4667 cases and 22,639 controls, found that DLBCL is associated with B-cell activating autoimmune diseases (odds ratio [OR] = 2.36, 95% confidence interval (CI) = 1.80 to 3.09), hepatitis C virus seropositivity (OR = 2.02, 95% CI = 1.47 to 2.76), family history of NHL (OR = 1.95, 95% CI = 1.54 to 2.47), and higher young adult body mass index (OR = 1.58, 95% CI = 1.12 to 2.23, for 35+ vs 18.5 to 22.4 kg/m^2^). Conversely, different potential presumptive protective factors have been proposed, such as higher sun exposure (OR = 0.78 and 0.80, 95% CI = 0.69 to 0.89 and 0.71 to 0.90), in two studies, and lifetime alcohol consumption (OR = 0.57, 95% CI = 0.44 to 0.75, for >400 g vs nondrinker) in one study. Vitamin D deficiency has been suggested as negative prognostic factor in patients with aggressive DLBCLs but was not found to be associated with dietary intake (OR = 1.03, 95% CI = 0.90 to 1.19), hence indicating that other factors rather than vitamin D may be involved [30,31]. More recent studies evaluating DLBCL risk, one using lipid trait variants in 2661 cases and 6221 controls found positive association with high density lipoproteins (OR = 1.14; 95% CI, 1.00–1.30), while another study evaluating height as variable; however, neither of them were significant after adjusting for multiple testing [10,18].

HLA-B (rs2523607) locus has been associated with DLBCL risk, initially described in a GWAS study with 3857 cases/7766 controls from European population (OR = 1.32; 95% CI = 1.21–1.44; *p* = 2.40 × 10^−10^) and then reported in 1124 patients and 3596 controls from Asian population (OR = 3.05; 95% CI = 1.32–7.05; *p* = 9.0 × 10^−3^), though not reaching genome-wide significance [32,33] (Table 1). Regarding pleiotropy of DLBCL with other diseases, a European population study found that variant rs10484561 (HLA-DQB1*01:01˜DQA1*01:01˜DQB1*05:01 extended haplotype, Linkage Disequilibrium (LD) r^2^ = 1.0) is associated with both DLBCL risk (OR = 1.36; 95% CI = 1.21–1.52; *p* = 1.40 × 10^−7^) and FL risk (OR = 1.64; 95% CI = 1.45–1.86; *p* = 5.0 × 10^−15^) using independent cohorts [34]. Additionally, a GWAS of 3857 DLBCL cases and 7666 controls used previously systemic lupus erythematosus (SLE) associated loci to evaluate the risk of DLBLC, finding HLA risk allele rs1270942. Another study evaluated multiple sclerosis (MS) and rheumatoid arthritis (RA) with DLBCL risk, but not genome-wide significance was reached [11,19]. Moreover, HLA homozygosity was found to be associated with increased DLBCL risk for HLA-B, HLA-C and HLA-DRB1 alleles among Europeans [35].

GWAS have been also widely used for non-HLA alleles associations, more remarkably susceptibility risk have been found in different studies for: *PVT1* (rs4733601 and rs13255292) in three different GWAS studies in which one also found to be associated with MS risk (*p* = 5 × 10^−8^); *EXOC2* (rs116446171) [11,31,32] and *CD86* (rs2681416 and rs9831894) [32,36]. *PVT1* is a non-coding RNA affecting MYC activation, a driver gene in lymphomas; *EXOC2* functions at the interface between host defense and cell death regulation and *CD86* is well known for its role in T-cell activation [32,37]. Other, implicated loci are 2p23.3 (*NCOA1*), 3p24.1 (*EOMES-AZI2*), 5q31.3 (*ARAP3*) and 3q27 (*BCL6-LPP*); interestingly the BCL6 has been vastly documented to be involved in B-cell lymphomagenesis due to its role as critical regulator of germinal centers and rs6773363 (*EOMES-AZI2)* is indirectly involved in the activation of the NF-κB signaling pathway [32,36,38]. Another study (399 DLBCL cases and 4243 controls) of Japanese population, identified risk for a variant within intron 3 of *CDC42BPB* (OR = 3.5; 95% CI = 2.13–5.88; *p* = 3.30 × 10^−7^), a gene with cell migration and cytoskeletal reorganization functions, a variant on *LNX2* (OR = 1.43; 95% CI = 1.23–1.67; *p* = 6.57 × 10^−6^), which indirectly mediates the NOTCH signaling and variant on POU6F2 (OR = 1.57; 95% CI = 1.32–1.88; *p* = 7.05 × 10^−7^), a transcriptional regulator [39]. In addition to PVT1, other overlapping risk variants for DLBCL and MS were rs1270942 (*RDBP*), rs3130557 (*PSORS1C1*), and rs2425752 (*NCOA5*) [11].

Another GWAS approach, using 491 DLBCL WGS data (31% discovery cohort; 69% validation cohort) and 1000 control WGS data, found NF-κB pathway activation by 3′ cis-regulatory mutations on *NFKBIZ* but only on ABC DLBCL subtype which was later correlated with increasing expression on different DLBCL cell lines when compared to the non-mutated ones. GCB subtype, on the other hand, was associated with poor overall survival for *FCGR2B* over expressing patients (HR = 2.18; *p* = 5.7 × 10^−3^) [40]. Furthermore, though it has not been fully explored, some studies have shown that the presence of activation-induced cytidine deaminase (AICDA) targeting motifs (WRC/GYW) within different point mutations, for example provoking induced translocations of PD-L1/PD-L2 with PIM1, TP63 and IGH or changes on the general mutational signatures across germinal center subtypes [40,41,42].

GWAS studies with survival data provided some evidence to finding potential prognostic or therapeutic targets in DLBCL, for example, a two-stage French study comprising four different cohorts in European population led to the discovery of two non-coding variants. The first one was rs7712513 at 5q23.2 (near *SNX2* and *SNCAIP*). The second one was rs7765004 at 6q21 (near *MARCKS* and *HDAC2*) that reached genome-wide significance for overall-survival association but not for progression free survival (PFS; Table 2). *SNX2* expression is reduced in human colorectal carcinoma and has been identified as a fusion partner of *ABL1* in B-cell acute lymphoblastic leukemia; meanwhile, *SNCAIP* has been only reported in medulloblastoma studies. On the other hand, *MARCKS* has been widely studied for its role in invasion, proliferation, and drug resistance within different types of cancers [28,43,44,45]. Another study using data recovered from the Genome Expression Omnibus (GEO) from 1804 DLBCL patients and performed a guilt-by-association analysis of only the 500 top-ranked CD20-associated gene probes. This study found *WEE1*, a replication checkpoint kinase that arrests cells at the G2/M checkpoint to give time for DNA repair, and *PARP1*, a repairing protein involved in high genomic instability and NF-kB activation, as potential candidates for DLBCL treatments. They further evaluated these targets using inhibiting drugs (AZD1775 for WEE1 and olaparib for PARP1) on different cell lines finding increased cytotoxic effects. Furthermore, a later study from the same group led to the discovery that combined WEE1 and anti-apoptotic protein inhibition enhances premature mitotic entry and DNA damage which may benefit genomic unstable DLBCL cells [17,46]. Moreover, there are over 20 clinical trials exploring adavosertib, the most potent and selective WEE1 inhibitor, as a single agent or in combination for different indications (clinicaltrials.gov, October 2020).

### 3.2. FL

Follicular lymphoma is an indolent B-cell malignancy with higher five-year survival than DLBCL, though a subset of tumors can transform into more aggressive forms of lymphomas. FL is characterized by variable clinical outcomes, multiple relapses, and risk associations that includes family history of NHL (OR = 1.99; 95% CI = 1.55 to 2.54) and greater body mass index (OR = 1.15; 95% CI = 1.04 to 1.27 per 5 kg/m(2) increase) [47,48,49]. Two different three-stage GWAS studies in European populations, found that variant rs10484561 is associated with FL risk (OR_1_ = 1.95; OR_2_ = 1.64; 95% CI_1_ = 1.72–2.22; 95% CI_2_ = 1.45–1.86; *p* < 1 × 10^−8^) which, in addition, was later found to be implicated in DLBCL risk and in complete linkage disequilibrium with the HLADRB1*01:01˜DQA1*01:01˜DQB1*05:01 haplotype (LD-r^2^ = 1.0). Additional HLA-DQB1 variants associated with FL risk were, rs7755224 and rs2647012. The first one was in complete LD with variant rs10484561 suggesting that the effect was related to this. The last one was found 962 base pairs away from rs10484561. However, they were not in LD (r^2^ < 0.1) and the association was protective and genome-wide significant after mutual adjustment (rs2647012-OR = 0.70; 95% CI = 0.67–0.78; *p* = 4 × 10^−12^; rs10484561-OR = 1.64; 95% CI = 1.45–1.86; *p* = 5 × 10^−15^), suggesting a totally different evolutionary origin [34,50]. This last variant was later found on additional studies in different populations (Caucasians and Chinese) at genome-wide significance [51,52]. Additional protective variants, found in another study with 699 cases and 2222 controls, were rs9275517 (OR = 0.63; 95% CI = 0.55–0.73; *p* = 4.03 × 10^−11^) and rs3117222 (OR = 0.66; 95% CI = 0.57–0.77; *p* = 1.45 × 10^−7^); furthermore, the second variant was correlated with higher *HLA-DPB1* expression in lymphoblastoid cell lines by using mRNA expression from MuTHER and Gen Cord datasets [53]. In 2014, Skibola et al. identified two variants within the HLA-II class to be significantly associated with increased FL risk (rs12195582-OR = 1.78; 95% CI = 1.68–1.88; *p* = 5.36 × 10^−100^; rs17203612-OR = 1.43; 95% CI = 1.32–1.57; *p* = 4.59 × 10^−16^) [54]. In addition to pleiotropy with DLBCL, HLA variants associated at genome-wide significance with FL has also been found for SLE, specifically two variants at HLA-DOB allele (rs1894406 and rs2071475) and one at HLA-DRB1 allele (rs9271775) [11]. In respect to homozygosity, HLA-DRB1 and HLA-DRQ1 alleles were found to be associated with increased FL risk [35].

A two-stage study with 238 FL cases and 1233 controls from United States found a variant in *TAP2* gene (rs241447) to be associated with increase FL risk (OR = 1.82; 95% CI = 1.46–2.26; *p* = 6.9 × 10^−8^) but also with DLBCL (189 cases) risk, though DLBCL being not at genome-wide significance. TAP2 is part of the multidrug resistance protein (MRP)/TAP subfamily of ATP-binding cassette (ABC) transporter, having an essential role for HLA class I protein loading on the cell surface and it is said that down-regulation or loss of function allows tumors to escape immune recognition [52]. One variant (rs6457327) near the psoriasis susceptibility locus (*PSORS1*) was found to be significantly associated to higher FL risk (OR = 1.69; 5% CI = 1.43–2.00; *p* = 4.7 × 10^−11^) among Europeans [55]. Skibola et al. recompiled information from 22 studies (4523 cases and 13,344 controls) from European populations and found five significant associated loci: rs6444305 (OR = 1.21; 95% CI = 1.14–1.28; *p* = 1.10 × 10^−10^) located in *LPP* which encodes a LIM domain containing protein that has cell adhesion, migration and proliferation roles and also found 836.4 kb upstream of *BCL6*; rs13254990 (OR = 1.18; 95% CI = 1.11–1.24; *p* = 1.06 × 10^−8^) located intronic to *PVT1,* a frequent translocation site in aggressive B-cell lymphomas; rs4938573 (OR = 1.34; 95% CI = 1.26–1.46; *p* = 5.79 × 10^−20^) located 12.6 kb upstream *CXCR5*, involved in B-cell migration; rs4937362 (OR = 1.19; 95% CI = 1.13–1.25; *p* = 6.76 × 10^−11^) located near *ETS1*, a transcription factor for B-cell differentiation; rs17749561 (OR = 1.34; 95% CI = 1.22–1.47; *p* = 8.28 × 10^−10^) located near *BCL2*, an anti-apoptotic oncogene. Furthermore, another interesting, though not genome-wide significant, rs2681416 variant (near *CD86*) showed increased risk of FL [54]. A Chinese study evaluating 792 cases and 1542 controls used additive genetic models adjusted with the false-positive rate probability to evaluate GWAS significance. This study found a variant on *IRF4* (rs872071), a crucial gene for B-cell development, to be associated with increased FL risk [51]. Moreover, this variant was found to be also associated with CLL risk in two additional European population studies [56,57]. Additionally, five more variants (*CFB, MSH5, TNXB, LOC649925,* and *UBE2L3*) on chromosome 6 were associated with FL and SLE risk [11].

ABC transporter variants associated with FL with worse PFS are *ABCA10* and *ABCA6* (rs10491178; HR = 3.17; 95% CI = 2.09–4.79; *p* = 5.24 × 10^−8^)which is in high LD (r2 > 0.8) with another variant within the binding site of a transcription factor, *PAX5,* that has been correlated with aggressive subsets of B-cell NHL. These results were found among Europeans along with a variant on *CD46* (rs2466571; HR = 0.73; 95% CI = 0.58–0.91; *p* = 6 × 10^−3^), *IL8* (rs4073; HR = 0.78; 95% CI = 0.62–0.97; *p* = 0.02), and *MTHFR* (rs1801131; HR = 0.59; 95% CI = 0.45–0.77; *p* = 1 × 10^−4^), albeit positively associated with event-free survival after adjusting for age, sex and population stratification. [47].

### 3.3. CLL

Another indolent lymphoma is CLL since the five-year survival rate is ~85% and it is characterized by a very rare incidence among Asian descendants compared to Caucasians and nearly double in males compared to females. Risk factors with CLL were previously identified to be family history of NHL (OR = 1.92; 95% CI = 1.42 to 2.61), hepatitis C virus infection (OR = 2.08; 95% CI = 1.23 to 3.49), and height (OR = 1.08, 95% CI = 1.00–1.17, *p* = 0.049) showing a slightly stronger trend among women (OR = 1.15, 95% CI: 1.01–1.31, *p* = 0.036). Conversely, immune function through allergy had a protective effect (OR = 0.87; 95% CI = 0.77 to 0.98) [10,58]. Additionally, an analysis of 13 cancer types including 49,492 cancer case patients and 34,131 control patients found that individuals with a high risk score for CLL were at an increased relative risk of DLBCL (RR = 1.12, 95% CI = 1.07 to 1.16) [59]. HLA associations to CLL were mainly reported for the expanded haplotype DRB4*01:01∼DRB1*07:01∼DQB1*03:03 in Caucasians (OR = 1.49; *p* = 1.79 × 10^−7^), African Americans (OR = 28.03; *p* = 2 × 10^−16^), and Hispanics (OR = 13.86; *p* = 9.59 × 10^−9^) and HLA-DRB4*0103 in a German study (RR = 2.74; *p* = 0.0025) [60,61]. Other study from Caucasian population, found five variants from which two were associated with increase disease risk at genome-wide significance, one located near *HLA-DRB5* (rs674317) and the other near *HLA-DQA1* (rs9272535) [62]. On the other hand, so far there are no indicators of HLA zygosity associations with CLL risk [35,63].

Because there have been several studies accessing CLL risk outside the HLA region, some variants have been validated across GWAS, for example variant rs17483466 (2q13; *ACOXL* and *BCL2L11*; p_1_ = 2.36 × 10^−10^;^,^ p_2_ = 5 × 10^−9^; p_3_ = 4 × 10^−17^), rs735665 (11q24.1; *GRAMD1B*; p_1_ = 3.78 × 10^−12^; p_2_ = 4 × 10^−24^) and rs210142 (6p21.31; *BAK1;* p_1_ = 9.47 × 10^−16^; p_2_ = 2.28 × 10^−16^) [56,64,65,66]. Some biologically interesting variants include one encoding a protein involved in the signal transduction downstream of Ras, *PCEF1,* which happens to reside within a strong enhancer element (rs2236256; OR = 1.23; *p* = 1.5 × 10^−10^); a telomer protecting protein, *POT1* (rs17246404; OR = 1.22; *p* = 3.40 × 10^−8^) [66]; a member of the tumor necrosis factor which is essential for the signaling cascade in apoptosis, *ACTA2*/*FAS* (rs4406737; OR = 1.27; *p* = 1.22 × 10^−14^); a lymphocyte’s apoptosis blocker, *BCL2* (rs4987855; OR = 1.47; *p* = 2.66 × 10^−12^) [65]; a member of the T-box gene family that regulates CD8^+^ T-cell differentiation and immunity, *EOMES* (rs9880772; p_1_ = 3 × 10^−11^; p_2_ = × 10^−9^; p_3_ = 2 × 10^−9^), which is also critical during Fas deficiency for lymphoproliferation [67,68,69]; a B-cell specific scaffold protein involved in B-cell antigen receptors, *BANK1* (rs71597109; OR = 1.17; *p* = 1.37 × 10^−10^); a master regulator of lymphocyte fate (B-cell vs T-cell) which is also involved in NOTCH pathway activation, *ZBTB7A* (rs7254272; OR = 1.17; *p* = 4.67 × 10^−8^) [68] and a regulator of the PI3K/Akt pathway, *NCK1* (rs11715604; *p* = 1.97 × 10^−8^) [69].

Other implicated loci by GWAS include: 2q33.1 (rs3769825; *CASP10/CASP8*; *p* = 2.5 × 10^−9^),2q37.1 (rs13397985; *SP140*; *p* = 5.40 × 10^−10^), 2q37.3 (rs757978; FARP2; OR = 1.39; *p* = 2.11 × 10^−9^), 3q25.2 (rs10936599; *MYNN*; *p* = 1.74 × 10^−9^), 4q25 (rs898518; *LEF1; p* = 4.24 × 10^−10^), 4q26 (rs6858698; *CAMK2D*; *p* = 3.07 × 10^−9^), 6p25.3 (rs872071 and rs9378805; *IRF4*; *p* = 1.91 × 10^−20^), 8q24.21 (rs2456449; *p* = 7.84 × 10^−10^), 11q24.1 (rs735665; *GRAMD1B*; *p* = 3.78 × 10^−12^), 12q24.13 (rs10735079; *OAS3*; *p* = 2.34 × 10^−8^), 15q23 (rs7176508; *DRAIC*; *p* = 8 × 10^−18^), 15q21.3 (rs7169431; *IRF8*; *p* = 4.74 × 10^−7^), 15q23 (rs7176508; *p* = 4.54 × 10^−12^), 16q24.1 (rs305061; *NEDD4* and *RFX7*; *p* = 3.60 × 10^−7^), and 19q13.32 (rs11083846; *PRKD2*; *p* = 3.96 × 10^−9^) [56,64,65,70,71]. Slager et al. found four additional *IRF8* variants associated to both decreased CLL risk and increased IRF8 expression (using lymphocytes cell lines data) which is opposite to the previous finding by Crowther et al. one year earlier [57,62]. *IRF4* and *IRF8* are a strong finding due to its role as key regulator of B-cell development, proliferation, and lymphogenesis; furthermore, associations for variant (rs872071) were also found for FL [51,70].

In spite of these findings several CLL associated risk variants have been also found for SLE, MS, and RA, only few have reached genome-wide significance; for example, a variant on gene *BCL2* (rs4987855) which has anti-apoptotic activity [11].

### 3.4. MZL

Marginal zone lymphoma, which comprises 10% of NHL cases, originate from marginal zone B cells present as three different types: extranodal MZL of mucosa-associated lymphoid tissue (EMZL) and splenic MZL (SMZL) and nodal MZL (NMZL) [64,65,72,73]. Risk factors for MZL include autoimmune conditions (EMZL OR = 6.40, 95% CI = 4.24–9.68; NMZL OR = 7.80, 95% CI = 3.32–18.33; SMZL OR = 4.25, 95% CI = 1.49–12.14), hepatitis C virus seropositivity (EMZL OR = 5.29, 95% CI = 2.48–11.28), self-reported peptic ulcers (EMZL OR = 1.83, 95% CI = 1.35–2.49),or family history NHL (NMZL OR = 2.82, 95% CI = 1.33–5.98). On the contrary, triglycerides levels were found as protective factor (OR = 0.90; 95% CI, 0.83–0.99) [18,66,74]. GWAS studies in MZL, which are less extensive. One study comprised 1281 cases and 7127 controls of European ancestry in which a variant in HLA-B allele (rs2922994; OR = 1.64; 95% CI = 1.39–1.92; *p* = 2.43 × 10^−9^) was found to be associated with increased MZL risk taken together with a variant on *BTNL2* (rs9461741; OR = 2.24, 95% CI = 1.64–3.07; *p* = 3.95 × 10^−15^), a gene involved lymphocyte activation and antigen presentation [64,72]. A second study assessing the role of HLA homozygosity in MZL risk (increased for HLA-B, HLA-C, and HLA-DRB1). A third study assessed the pleiotropy with SLE (*RDBP, PSORS1C1,* and *HLA-DQA1*) and RA (*CDH8*) [11].

### 3.5. PCNSL

Recognized as mature post-germinal B cells (ABC subtype like), the central nervous system (CNS) DLBCL represents only ≤1% of all lymphomas and approximately 2% of all primary CNS tumors; furthermore, 95% of tumors have a comparative histology with systemic DLBCL [67,75]. Since the blood brain barrier impedes R-CHOP treatment, high dose methotrexate (HD-MTX, >3 g/m^2^) based regimens are the gold standard for PCNSL patients resulting in a five year survival rate of 30% and high risk of clinical neurotoxicity specially in patients > 60 [68,76]. Furthermore, PCNSL is less frequently associated with any atopic disorder (OR = 0.54, 95% CI = 0.33 to 0.87), but it is strongly associated with a family history of NHL (OR = 4.11, 95% CI = 1.58 to 10.66) and less clearly with lifetime cigarette exposure (OR = 1.51, 95% CI = 0.83 to 2.74, for 1–10 pack-years vs. nonsmoker) [30]. The only study that has reported associations between genetic variants and PCNSL risk, evaluated 475 cases and 1134 controls from French population. This study found one variant at loci 6p25.3 (rs116446171; *EXOC2; p* = 1.95 × 10^−13^) previously found to be associated with DLBCL risk, other at loci 3p22.1 (rs41289586, *ANO10*, *p* = 2.17 × 10^−8^) and one strongly associated at HLA allele (rs2395192; between HLA-DRA and HLA-DRB5; *p* = 1.81 × 10^−7^). *ANO10* is a calcium-activated chloride channel transmembrane protein that might be involved in the innate immune defense and indirect activation of the Ras/Raf/MEK/ERK signaling pathway which affects cell proliferation [67,69,75,77].

## 4. Conclusions

Initially data suggested increased risk of any DLBCL, FL, CLL, MZL, or PCNSL if family history of NHL was present, though specific genetic attributions for specific risk or prognosis had been lacking [30,49,58,74]. The decreasing cost and bioinformatics limitations for NGS and GWAS have augmented the ability to detect the genetic risk for NHL etiologies, pleiotropy with other diseases and, more importantly, clinical applications. Despite that many HLA alleles have been found to be not only associated to specific lymphoma subtypes but also within subtypes (DLBCL and FL) or with autoimmune diseases (MS, SLE, or RA), today they remain just informative for prognosis since no clinical use has been made [7,11,14]. Peptide diversity reduction can increase tumoral escape from immune surveillance, which can be partially a consequence of HLA homozygosity, which was found to be a risk factor for most of the reviewed lymphomas [35,63].

On the other hand, GWAS findings outside HLA loci have led to the discovery of B-cell NHLs shared genetic risk with autoimmune diseases leading to finding of genes involved in cell cycle, apoptosis and telomere length, though studies are limited and total risk is still modest at genome wide-significance.

Taken together that hepatitis seropositivity is associated with DLBCL, CLL and MZL risk and that AICDA promiscuous off-target activity, highly present in lymphomas and induced by viral infection, can provoke important alterations (example translocations of PD-L1/PD-L2 with *PIM1*, *TP63,* and *IGH* loci). Further efforts should be made to find correlations between these variables [41,80,81,82]. Future efforts should also be directed to extend studies among non-Caucasian populations, in order to clarify differences in susceptibility variants, and among B-cell NHLs subtypes since most studies have focused on DLBCL. Furthermore, there is an unmet need to translate theoretical information into clinical practice which has been done, for example, with the use of adavosertib, an WEE1 inhibitor, to increase response in DLBCL patients. In line with this, the incorporation of single-cell sequencing technology can help identify B-cell stages (dark/light zone) and cell cycle phases to further amplify the possibilities for therapy options [17,46,83].

## Figures and Tables

**Table 1:** Risk associations summary for diffuse large B-cell lymphoma (DLBCL), follicular lymphoma (FL), chronic lymphocytic leukemia (CLL), marginal zone lymphoma (MZL) and primary central nervous system lymphoma (PCNSL) with different loci identified by genome-wide association studies (GWAS).

Study	Year	Race/Ethnicity	#Cases/#Controls	SNP/Alteration	Chr	Gene(s)	OR (95% CI)	*p*-Value	Reference
DLBCL risk	2009	European	783/3377	rs6457327	6p21.33	PSORS1 *	1.69 (1.43–2.00)	7.0 × 10^−5^	[54]
	2011	European	1592/6581	rs10484561	6p21.32	*HLA-DQB1*	1.36 (1.21–1.52)	1.4 × 10^−7^	[34]
	2011	Asian	399/4243	rs751837	14q32	*CDC42BPB*	3.5 (2.127–5.88)	3.3 × 10^−7^	[39]
				rs7097	13q12	*LNX2*	1.437 (1.23–1.67	6.5 × 10^−6^	
				rs4551233	7	*POU6F2*	1.57 (1.32–1.88)	7.05 × 10^−7^	
				rs4443228	4	*--*	2.43 (1.70–3.45)	7.03 × 10^−7^	
	2013	Asian	1328/6930	rs6773854	3q27	BCL6 *, LPP *	1.47 (1.32–1.65)	1.14 × 10^−11^	[69,78]
	2014	European	3857/7666	rs2523607	6p21.33	*HLA-B*	1.32 (1.21–1.44)	2.40 × 10^−10^	[32]
				rs116446171	6p25.3	EXOC2 *	2.20 (1.87–2.59)	2.33 × 10^−21^	
				rs79480871	2p23.3	*NCOA1*	1.34 (1.21–1.49)	4.23 × 10^−8^	
				rs13255292	8q24.21	*PVT1*	1.22 (1.15–1.29)	9.98 × 10^−13^	
				rs4733601			1.18 (1.11–1.25)	3.63 × 10^−11^	
				rs79464052	5q31.3	*ARAP3*	1.34 (1.21–1.49)	5.57 × 10^−8^	
				rs2681416	3q13.33	*CD86*	1.16 (1.10–1.23)	8.17 × 10^−8^	
	2015	Asian	1124/3596	rs116446171	6p25.3	EXOC2 *	2.04 (1.63–2.56)	3.9 × 10^−10^	[33]
				rs13255292	8q24.21	*PVT1*	1.34 (1.19–1.52)	2.1 × 10^−6^	
				rs2523607	6p21.33	*HLA-B*	3.05 (1.32–7.05	9 × 10^−3^	
	2018	European	3617/8753	Homozygosity	6p21.33	*HLA-B, HLA-C*	1.31 (1.06–1.60)	8 × 10^−4^	[35]
	2018			Homozygosity		*HLA-DRB1*	2.10 (1.24–3.55)	1 × 10^−4^	
	2019	European	5662/9237	rs9831894	3q13.33	CD86 *, ILDR1 *	0.83	3.62 × 10^−13^	[36]
			5510/12,817	rs6773363	3p24.1	EOMES *, AZI2 *	1.20	2.31 × 10^−12^	
DLBCL-SLE	2017	European	3857/7666	rs4810485	20q13	*CD40*	1.09 (1.02–1.16)	0.013	[19]
				rs1270942	6p21.33	*HLA*	1.17 (1.01–1.36)	0.036	
	2019	European	3617/46,436	rs1270942	6	*RDBP*	NA	5 × 10^−8^	[11]
				rs3130557	6	*PSORS1C1*	NA		
				rs4733601	8	*PVT1*	NA		
DLBCL-MS				rs2425752	20	*NCOA5*	0.91	3.4 × 10^−2^	
FL-risk	2009	European	645/3377	rs6457327	6p21.33	PSORS1 *	1.69 (1.43–2.00)	4.7 × 10^−11^	[54]
	2010	European	1465/6958	rs10484561	6p21.32	*HLA-DQB1*	1.95 (1.72–2.22)	1.12 × 10^−29^	[50]
				rs7755224	6p21.32		2.07 (1.76–2.42)	2.0 × 10^−19^	
	2011	European	1428/6761	rs10484561	6p21.32	*HLA-DQB1*	1.64 (1.45–1.86)	5 × 10^−15^	[34]
				rs2647012	6p21.32		0.70 (0.67–0.78)	4 × 10^−12^	
	2012	Caucasians	699/2222	rs9275517	6p21.32	HLA-DRB1 *	0.63 (0.55–0.73)	4.0 × 10^−11^	[53]
				rs3117222		HLA-DPB1 *	0.66 (0.57–0.77)	1.45 × 10^−7^	
	2013	Caucasians	238/1233	rs2647012	6p21.32	*HLA-DQB1*	0.56 (0.45–0.69)	8.03 × 10^−8^	[52]
				rs241447	6p21.3	*TAP2*	1.82 (1.46–2.26)	6.9 × 10^−8^	
		Asian	792/1542	rs2647012	6p21.32	*HLA-DQB1*	1.20 (1.03–1.39)	0.018	[51]
				rs872071	6p25.3	*IRF4*	1.20 (1.05–1.38)	0.009	
	2014	European	4523/13,344	rs12195582	6p21.32	HLA-DRB5	1.78 (1.68–1.88)	5.36 × 10^−100^	[54]
				rs17203612	6p21.32	HLA-DRB1	1.43 (1.32–1.57)	4.59 × 10^−16^	
				rs4938573	11q23.3	CXCR5 *	1.34 (1.26–1.43)	5.79 × 10^−20^	
				rs4937362	11q24.3	ETS1 *	1.19 (1.13–1.25)	6.76 × 10^−11^	
				rs6444305	3q28	*LPP*	1.21 (1.14–1.28)	1 × 10^−10^	
				rs17749561	18q21.33	BCL2 *	1.34 (1.22–1.47)	8.28 × 10^−10^	
				rs13254990	8q24.21	PVT1 *	1.18 (1.11–1.24)	1.06 × 10^−8^	
				rs3751913	17q25.3	*CYBC1*	1.23 (1.14–1.33)	2.24 × 10^−7^	
				rs2681416	3q13.33	*CD86*	1.16 (1.09–1.22)	2.33 × 10^−7^	
				rs11082438	18q12.3	*SLC14A2*	1.33 (1.19–1.48)	4.01 × 10^−7^	
	2018	European	2686/8753	Homozygosity	6p21.32	*HLA-DRB1*	1.54 (1.31–1.82)	1 × 10^−4^	[35]
				Homozygosity	6p21.32	*HLA-DQB1*	1.42 (1.23–1.65)	1 × 10^−4^	
FL-DLBCL	2011	European	1428/6581	rs2647012	6p21.32	*HLA-DQB1*	1.36	1.4 × 10^−7^	[50]
FL-SLE	2019	European	2686/46,436	rs1015166	6	*TAP2*	NA	5 × 10^−8^	[11]
				rs1894406	6	*HLA-DOB*	NA		
				rs2071475	6	*HLA-DOB*	NA		
				rs2072634	6	*CFB*	NA		
				rs2293861	6	*MSH5*	NA		
				rs7774197	6	*TNXB*	NA		
				rs9271775	6	*HLA-DRB1*	NA		
				rs4938573	11	*LOC649925*	NA		
				rs7444	22	*UBE2L3*	NA		
CLL-risk	2001	European	101/157	---	6	HLA-DRB4 *0103	2.74	2.5 × 10^−3^	[60]
	2008	European	1529/3115	rs17483466	2q13	*ACOXL, BCL2L11*	1.39 (1.25–1.53)	2.36×10^−10^	[55]
				rs13397985	2q37.1	SP140 *, SP110 *	1.41 (1.26–1.57)	5.40 × 10^−10^	
				rs872071	6p25.3	*IRF4*	1.54 (1.41–1.69)	1.91 × 10^−20^	
				rs9378805	6p25.3	*IRF4*	1.51 (1.38–1.65)	4.62 × 10^−19^	
				rs735665	11q24.1	*GRAMD1B*	1.45 (1.31–1.61)	3.78 × 10^−12^	
				rs7176508	15q23	*---*	1.37 (1.26–1.50)	4.54 × 10^−12^	
				rs11083846	19q13.32	*PRKD2*	1.35 (1.22–1.49)	3.96 × 10^−9^	
	2010	European	824/850	rs872071	6p25.3	*IRF4*	1.42 (1.23–1.63)	9.96 × 10^−7^	[56]
				rs735665	11q24.1	*GRAMD1B*	1.59 (1.34–1.88)	1.23 × 10^−7^	
	2010		2503/5789	rs757978	2q37.3	*FARP2*	1.39	2.11 × 10^−9^	[62]
				rs2456449	8q24.21	*---*	1.26	7.84 × 10^−10^	
				rs7169431	15q21.3	IRF8 *	1.36	4.74 × 10^−7^	
				rs305061	16q24.1	NEDD4 *, RFX7 *	1.22	3.60 × 10^−7^	
	2011	Caucasians	690/1295	rs305077	16q24.1	*IRF8*	0.66 (0.57–0.77)	3.37 × 10^−8^	[62]
				rs391525			0.64 (0.55–0.74)	3.16 × 10^−9^	
				rs2292982			0.65 (0.56–0.75)	6.48 × 10^−9^	
				rs2292980			0.66 (0.56–0.76)	1.89 × 10^−8^	
				rs615672	6p21.3	*HLA-DRB5*	1.42 (1.22–1.67)	1.29 × 10^−5^	
				rs674313			1.69 (1.41–2.01)	6.92 × 10^−9^	
				rs502771			1.61 (1.36–1.91)	5.58 × 10^−8^	
				rs9272219		*HLA-DQA1*	1.59 (1.34–1.90)	1.84 × 10^−7^	
				rs9272535			1.61 (1.35–1.92)	9.31 × 10^−8^	
	2012	European/American	1982/5778	rs210142	6p21.33	*BAK1*	1.40 (1.25–1.57)	9.47 × 10^−16^	[64]
	2012	European/American	1196/2410	rs210142	6p21.33	*BAK1*	0.73 (0.68–0.79)	2.28 × 10^−16^	[79]
	2013	European	3100/7667	rs4406737	10q23.31	ACTA2 *, FAS *	1.27 (1.19–1.33)	1.22 × 10^−14^	[65]
				rs4987855	18q21.33	*BCL2*	1.47 (1.32–1.61)	2.66 × 10^−12^	
				rs4987852			1.41 (1.27–1.56)	7.76 × 10^−11^	
				rs7944004	11p15.5	C11orf21, * TSPAN32 *	1.20 (1.13–1.27)	2.15 × 10^−10^	
				rs898518	4q25	*LEF1*	1.20 (1.14–1.27)	4.24 × 10^−10^	
				rs3769825	2q33.1	*CASP10, CASP8*	1.19 (1.12–1.25)	2.50 × 10^−9^	
				rs1679013	9p21.3	*CDKN2B-AS1*	1.19 (1.12–1.27)	1.27 × 10^−8^	
				rs4368253	18q21.32	*PMAIP1*	1.19 (1.12–1.27)	2.51 × 10^−8^	
				rs8024033	15q15.1	*BMF*	1.22 (1.15–1.30)	2.71 × 10^−10^	
				rs3770745	2p22.2	QPCT *, PRKD3 *	1.24 (1.15–1.33) 1.68 × 10^−8^	1.68 × 10^−8^	
				rs13401811	2q13	ACOXL *, BCL2L11 *	1.41 (1.30–1.52)	2.08 × 10^−18^	
	2014	Europeans	3748/8574	rs10735079	12q24.13	*OAS3*	1.18 (1.12–1.26)	2.34 × 10^−8^	[71]
	2014	Europeans	2883/8350	rs2236256	6q25.2	*IPCEF1*	1.23 (1.15–1.30)	1.5 × 10^−10^	[66]
				rs10936599	3q26.2	*MYNN*	1.26 (1.17–1.35)	1.74 × 10^−9^	
				rs6858698	4q26	*CAMK2D*	1.31 (1.20–1.44)	3.07 × 10^−9^	
				rs17246404	7q31.33	*POT1*	1.22 (1.14–1.31)	3.40 × 10^−8^	
				rs1439287	2q13	*ACOXL*	1.37	5 × 10^−15^	
				rs13397985	2q37.1	*SP140*	1.43	5 × 10^−13^	
				rs872071	6p25.3	*IRF4*	1.39	3 × 10^−16^	
				rs735665	11q24.1	*GRAMD1B*	1.64	4 × 10^−24^	
				rs7176508	15q23	*DRAIC*	1.42	8 × 10^−18^	
				rs1044873	16.q24.1	*IRF8*	1.29	1 × 10^−9^	
	2014	Caucasian	3616/50,000	---	6	HLA-DRB4 *01:01 *∼* DRB1 *07:01 *∼* DQB1 *03:03	1.49	1.79 × 10^−7^	[60]
		African-American	413/50,000		6		28.03	2 × 10^−16^	
		Hispanic	97/50,000		6		13.86	9.59 × 10^−9^	
	2016	Europeans	5058/13,197	rs9880772	3p24.1	*EOMES*	1.19 (1.13–1.25)	2.5 × 10^−11^	[67]
				rs73718779	6p25.2	*SERPINB6*	1.26 (1.16–1.36)	1.97 × 10^−8^	
				rs9815073	3q28	*LPP*	1.18 (1.11–1.25)	3.26 × 10^−8^	
				rs9308731	2q13	*BCL2L11*	1.19 (1.13–1.26)	1 × 10–^11^	
				rs10028805	4q24	*BANK1*	1.16 (1.10–1.22)	7.19 × 10^−8^	
				rs1274963	3p22.2	*CSRNP1*	1.18 (1.11–1.25)	2.12 × 10^−7^	
	2017	Europeans	6200/17,598	rs34676223	1p36.11	*MDS2*	1.19 (1.14–1.25)	5.04 × 10^−13^	[68]
				rs41271473	1q42.13	*RHOU*	1.19 (1.13–1.26)	1.06 × 10^−10^	
				rs71597109	4q24	*BANK1*	1.17 (1.11–1.22)	1.37 × 10^−10^	
				rs57214277	4q35.1	MYL12BP2 *, LINC02363 *	1.13 (1.08–1.18)	3.69 × 10^−8^	
				rs3800461	6p21.31	*ILRUN*	1.20 (1.13–1.28)	1.97 × 10^−8^	
				rs61904987	11q23.2	TMPRSS5 *, DRD2 *	1.24 (1.16–1.32)	2.46 × 10^−11^	
				rs1036935	18q21.1	AC105227.1 *, AC105227.2 *	1.15 (1.10–1.21)	3.27 × 10^−8^	
				rs7254272	19p13.3	ZBTB7A *, MAP2K2 *	1.17 (1.10–1.23)	4.67 × 10^−8^	
				rs140522	22q13.33	*ODF3B*	1.15 (1.10–1.20)	2.7 × 10^−9^	
	2017	Europeans	1842/7324	rs11715604	3q22	*NCK1*	NA	1.97 × 10^−8^	[69]
				rs131821	22q13.33	*NCAPH2*	NA	7.49 × 10^−8^	
CLL-SLE	2019	European	2492/46,436	rs10028805	4	*BANK1*	NA	5 × 10^−8^	[11]
				rs1270942	6	*RDBP*	NA		
				rs17587	6	*PSMB9*	NA		
				rs3130557	6	*PSORS1C1*	NA		
				rs4987855	18	*BCL2*	NA		
				rs1439112	2	*MGAT5*	0.88	4.7 × 10^−2^	
				rs10936599	3	*MYNN, ACTRT3, TERC, LRRC34*	0.86	2.7 × 10^−2^	
				rs1317082	3			1.5 × 10^−2^	
				rs13069553	3			1.07 × 10^−2^	
				rs7621631	3			1.8 × 10^−2^	
				rs10069690	5	*TERT*	1.16	3.06 × 10^−2^	
CLL-MS				rs140522	22	*ODF3B*	0.90	4.32 × 10^−4^	
				rs6793295	3	*LRRC34*	0.90	1.24 × 10^−2^	
CLL-RA				rs3731714	2	*CASP10, PPIL3, CFLAR*	0.87	4.69 × 10^−2^	
MZL-risk	2015	European	1281/7127	rs2922994	6p21.32	*HLA-B*	1.64 (1.39–1.92)	2.43 × 10^−9^	[63]
				rs9461741	6p21.32	*BTNLA*	2.24 (1.64–3.07)	3.95 × 10^−15^	
	2018	European	741/8753	Homozygosity	6p21.33	*HLA-B*	1.34 (1.01–1.78)	0.012	[35]
				Homozygosity	6p21.33	*HLA-C*	1.33 (1.04–1.70)		
				Homozygosity	6p21.33	*HLA-DRB1*	1.45 (1.05–1.91)		
MZL-SLE	2019	European	741/46,436	rs1270942	6	*RDBP*	NA	5 × 10^−8^	[11]
				rs3130557	6	*PSORS1C1*	NA		
				rs532098	6	*HLA-DQA1*	NA		
MZL-RA				rs16947122	12	*FBXW8, HRK, TESC*	1.86	3.35 × 10^−2^	
				rs1364229	16	*CDH8*	1.35	1.10 × 10^−3^	
				rs7192064	16	*CDH8*	0.76	4.36 × 10^−2^	
				rs2131402	16	*CDH8*	0.75	1.01 × 10^−2^	
PCNSL-risk	2013	European	475/1134	rs41289586	3p22.1	*ANO10*	3.82 (2.39–6.09)	2.17×10^−8^	[66]
				rs116446171	6p25.3	EXOC2 *	4.99 (3.26–7.65)	1.95 × 10^−13^	
				rs2395192	6p21	HLA-DRA *, HLA-DRB5 *	1.51 (1.29–1.76)	1.81×10^−7^	

* Closest related gene; SLE: *lupus* erythematosus; MS: multiple sclerosis; RA: rheumatoid arthritis; NA: not available.

**Table 2:** Associations of different loci by GWAS with survival for DLBCL and FL.

Study	Year	Race/Ethnicity	# Cases	SNP/Alteration	Chr	Gene(s)	HR (95% CI)	*p*-Value	Outcome	Reference
DLBCL	2015	European	1537	rs7712513	5q23.2	SNX2 *, SNCAIP *	1.49 (1.29–1.72)	3.53 × 10^−8^	↓ OS	[28]
							1.39 (1.23–1.57)	2.08 × 10^−7^	↓ PFS	
				rs7765004	6q21	MARCK *, HDACS2 *	1.47 (1.27–1.71)	5.36 × 10^−7^	↓ OS	
							1.38 (1.22–1.57)	7.09 × 10^−7^	↓ PFS	
	2018	European	210	---	1q23.3	*FCGR2B*	2.18	5.7 × 10^−3^	↓ OS	[40]
FL	2014	European	586	rs10491178	17q24	ABCA10 *, ABCA6 *	3.17 (2.09–4.79)	5.24 ×10^−8^	↓ PFS	[47]
				rs2466571	1q32.2	*CD46*	0.73 (0.58–0.91)	6 × 10^−3^	↑ EFS	
				rs4073	4q13.3	*IL8*	0.78 (0.62–0.97)	0.02	↑ EFS	
				rs1801131	1p36.22	*MTHFR*	0.59 (0.45–0.77)	1 × 10^−4^	↑ EFS	

* Closest related gene; OS: overall survival; PFS: progression free survival; ↓: inferior; EFS: event free survival; ↑: superior.

## Abbreviations

NHL	Non-Hodgkin’s lymphoma
HL	Hodgkin’s lymphoma
DLBCL	Diffuse large B-cell lymphoma
FL	Follicular lymphoma
CLL	Chronic lymphocytic leukemia
MZL	Marginal zone lymphoma
PCNSL	Primary central nervous system lymphoma
NGS	Next generation sequencing
GWAS	Genome-wide association studies
HLA	Human leukocyte antigen
MHC	Major histocompatibility complex
NK	Natural killer
R-CHOP	Rituximab combined with cyclophosphamide, doxorubicin, vincristine, and prednisolone treatment
GCB	Germinal center B-cell
ABC	Activated B-cell
OR	Odds ratio
CI	Confidence interval
LD	Linkage disequilibrium
SLE	*Lupus erythematosus*
MS	Multiple sclerosis
RA	Rheumatoid arthritis
AICDA	Activation-induced cytidine deaminase
GEO	Genome Expression Omnibus
ABC	ATP-binding cassette
EMZL	Extranodal MZL of mucosa-associated lymphoid tissue
HD-MTX	High dose methotrexate
